# Epidemiology of hip fracture in Botswana

**DOI:** 10.1007/s11657-021-00885-x

**Published:** 2021-02-07

**Authors:** M. Kebaetse, S. Nkhwa, M. Mogodi, J. Masunge, Y. P. Gureja, M. Ramabu, T. Mmopelwa, I. Sharif, A. Orford, H. Johansson, N. C. Harvey, E. V. McCloskey, J. A. Cauley, J. A. Kanis

**Affiliations:** 1 Faculty of Medicine, University of Botswana, Gaborone, Botswana; 2 Princess Marina Hospital, Gaborone, Botswana; 3 Gaborone Private Hospital, Gaborone, Botswana; 4 Bokamoso Private Hospital, Gaborone, Botswana; 5 Centre for Metabolic Bone Diseases, University of Sheffield, Sheffield, UK; 6 Mary McKillop Institute for Health Research, Australian Catholic University, Melbourne, Australia; 7 MRC Lifecourse Epidemiology Unit, University of Southampton, Southampton, UK; 8 Mellanby Centre for Musculoskeletal Research, Department of Oncology and Metabolism, University of Sheffield, Sheffield, UK; 9 Department of Epidemiology, Graduate School of Public Health, University of Pittsburgh, Pittsburgh, PA USA

**Keywords:** Epidemiology, Hip fractures, Lifetime risk, Botswana, Population studies, Prevalence

## Abstract

***Summary*:**

A retrospective population-based survey in the Republic of Botswana determined the incidence of fractures at the hip over 3 years. The estimated number of such fractures nationwide for 2020 was 103 and is predicted to increase.

**Objective:**

This article describes the epidemiology of hip fractures in the Republic of Botswana.

**Methods:**

A retrospective patient chart review was conducted to identify from hospital registers the number of patients diagnosed with hip fracture in 2009, 2010, and 2011. Age- and sex-specific incidence of hip fracture was determined from which lifetime probabilities and future projections for hip fracture were calculated.

**Results:**

The incidence of hip fracture was low and comparable to rates reported from Tunisia. The remaining lifetime risk of hip fracture at the age of 50 years in men and women was 1.4 and 1.1%, respectively. The incidence of hip fracture suggested that the estimated number of hip fractures nationwide in persons over the age of 50 years for 2020 was 103 and is predicted to increase by more than threefold to 372 in 2050.

**Conclusion:**

The hip fracture rates can be used for healthcare planning. Additionally, these data can be used to create a FRAX model to help guide decisions about treatment.

## Introduction

At present, there is no information about osteoporosis and fractures in Botswana. Indeed, information from the African continent is scarce and until recently was outdated or of poor quality [1–6]. Although fracture rates have been measured in the Black population of the United States [7], there is little information to determine whether these are comparable to rates in Africa. Hip fractures are the international barometer of the impact of osteoporosis because of their severe socioeconomic costs and because they are more easily documented than other fractures and can be used to compare rates in different countries [8]. The objective of the present report is to summarise the work undertaken to document rates of hip fracture by age and sex in the Republic of Botswana.

## Methods

The Republic of Botswana is a landlocked country in Southern Africa. It is bordered by South Africa to the south and southeast, Namibia to the west and north, and Zimbabwe to the northeast. Its border with Zambia to the north near Kazungula is poorly defined. Botswana has an area of 581,730 km^2^ (224,607 square miles) with a population estimated at just over 2.3 million people [9]. The Tswana are the majority ethnic group, making up approximately 79% of the population, followed by Kalanga at 11% and the San (Basarwa) at 3%. Another 7% is made up of a number of other smaller Southern African ethnic groups, as well as Indians and people of European descent [9].

The primary objective of the present study was to determine the incidence of hip fracture in Botswana from January 2009 to December 2011. A retrospective patient chart review was conducted to identify and document the number of patients diagnosed with any fracture recorded and classified as a hip fracture or with ICD-10 codes for femoral head (S72.05), femoral neck (S72.0), per-trochanteric (S72.1), and subtrochanteric (S72.2) fractures.

A structured case report form was developed and a team of 4 researchers (two orthopaedic surgeons, a public health specialist, and a rehabilitation scientist) trained a total of 10 research assistants on the purpose of the study, proper chart review, hip fracture identification, and data collection and documentation. Each research assistant had a degree in biology or a related field. The inter-rater reliability of the data collection was determined by extracting data from the first 20 charts at one site using two independent research assistants. Any discrepancies in fracture classification were reviewed with the study team before any further data collection could continue.

Data were collected from a total of eight research sites, comprising two private hospitals located in the capital city; three tertiary-level public hospitals located in the southern, central, and northern parts of the country; and three private insurance companies. At the time of the study, these hospitals were the only centres in the country that treated hip fractures. The insurance companies were included to capture any other fracture that could have been missed at the hospitals or may have been treated outside the country.

Key terms (hip fracture, femoral fracture, and femur) were used to generate a list of patients with potential hip fractures from electronic and paper registries; the non-electronic registries were from emergency and operating rooms and surgical, oncology, and paediatric wards. The Integrated Patient Management System (IPMS), a national electronic database system for public hospitals, was searched to expand the lists for public hospitals. For the private sector, a list of patients with potential hip fractures was created from an electronic registry using all patients under the “lower limb injuries” category.

All charts were screened for the hip fracture diagnoses. The diagnosis in each patient was established from at least two sources of information that included radiology reports, digital radiology files, surgical ward notes and postoperative theatre notes, and discharge summaries. Information on each patient was recorded onto a structured case report form that included data on age, sex, site of fracture, degree of trauma, and ethnicity. Additional variables of interest were patient’s cause of injury, history and cause of previous hip fractures, HIV/AIDS status, and type of malignancy, if present. For quality assurance, the primary investigator reviewed all charts at the private hospitals and approximately 50% of randomly selected charts at the public hospitals.

Fracture cases were recorded from the age of 40 years since this is the age from which FRAX is used to calculate fracture probabilities. Multiple admissions by the same patient for the same fracture were excluded to avoid duplication. For the present analysis, we included patients irrespective of the degree of trauma. The reason for their inclusion is that classification of high and low energy fractures is inconsistent and arbitrary. Additionally, high-trauma and non-trauma fractures show similar relationships with low BMD and future fracture risk [10–12]. We also excluded 43 cases of acetabular fractures. We included patients characterised as white, mixed race, or Asian as they are recognised minorities in Botswana.

Incidence was computed by age and sex in 5-year age intervals using the population demography obtained from Statistics Botswana for 2011 [13]. Additionally, future projections were estimated up to 2050 assuming that the age- and sex-specific incidence remained stable. Population demography was taken from the United Nations using the medium variant for fertility [14]. For other major osteoporotic fractures (clinical spine, forearm, and humeral fractures), it was assumed that the age- and sex-specific ratios of these fractures to hip fracture risk found in Sweden were comparable to those in Botswana. This assumption has been used for many of the FRAX models with incomplete epidemiological information. Available information suggests that the age- and sex-stratified pattern of fracture is very similar in the Western world, Australia, and Eastern Europe [15–18].

The incidence of hip fracture was compared with other estimates from the region. The data for Morocco, Tunisia, American Blacks, and South African Blacks were those used in the current FRAX models. That of sub-Saharan Africa is an unpublished meta-analysis of available data for the region [1–5]. We also computed the remaining lifetime probability of hip fracture from the age of 50 years for men and women, as described by Kanis et al. [19, 20]. In the present analysis, values for Botswana were compared with those of China (with and without inclusion of Hong Kong), Canada, Denmark, Finland, France, Greece, Hungary, Moldova, Poland, Portugal, Romania, Russia, Spain, Sweden, Turkey, Ukraine, United Kingdom, and the United States [21] with more recent addition of South Africa [6].

## Results

A total of 435 hip fractures (196 in men, 239 in women) were recorded for the 3-year period from 2009 to 2011. The large majority (94.7%) of cases were Black Africans, 4.1% were Whites, and 0.9% were Asians. The most common site of hip fracture was at the femoral neck (55.4%) followed by per-trochanteric fractures (36.6%).

There were slightly more fractures in women than in men (239 vs. 196, respectively). The crude annual incidence was, however, close to equality (33.2 and 33.4/100,000, respectively). There were 39 high energy fractures coded in men and 10 in women. When these were excluded, the crude incidence was 26.6 and 32.0/100,000 in men and women, respectively, giving a female/male ratio of 1.20.

Hip fracture incidence increased with the age up to the age of 90 years in both men and women (Table 1).

**Table 1:** Population at risk (2011), number of hip fractures (2009–2011), and annual hip fracture incidence (per 100,000) with 95% confidence intervals in men and women from Botswana

Age	Number of hip fractures		Population		Men		Women	
(Years)	M	F	M	F	Incidence	95%CI	Incidence	95%CI
40–44	16	8	48767	50530	11	6–18	5	2–10
45–49	12	11	37881	44380	11	6–19	8	4–15
50–54	9	5	29742	36620	10	5–19	5	2–11
55–59	15	6	24368	29681	21	12–34	7	3–15
60–64	9	6	17344	20240	17	8–33	10	4–22
65–69	23	14	12243	15504	63	40–94	30	16–51
70–74	11	14	9464	12797	39	19–69	37	20–61
75–79	17	35	6968	10924	81	47–130	107	74–149
80–84	40	45	4875	8344	274	195–373	180	131–241
85–89	20	39	2825	5422	236	144–365	240	170–328
90–94	13	32	1377	2544	315	167–539	419	287–592
95+	11	24	842	1818	436	217–779	440	282–655

**Fig. 1:**
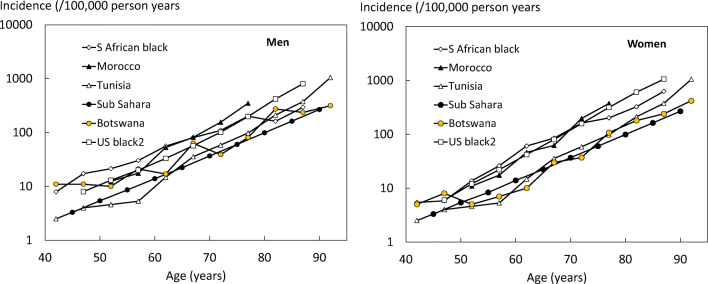
The incidence of hip fracture in men (left panel) and women (right) in Botswana compared with other estimates in Africa and African Americans. Note the logarithmic scale

The incidence of hip fracture is compared with other estimates from the region in Fig. 1. The logarithmic plot allows the slope of the logarithmic data and its elevation (intercept) with age to be visualised. The slope and elevation for the data from Botswana were very similar to that for Tunisia and sub-Saharan Africa. In contrast, the elevation in data for Morocco, Blacks from South Africa, and Blacks from the United States was higher than that for Botswana. The effect was more marked for women than for men.

Assuming that the fracture rates were representative for the whole country and based on the UN estimates of the population for 2020 (4.63 million, 608 thousand age 50 years or more), the annual number of hip fractures in men and women age 50 years or older in Botswana in 2020 was estimated at 103. The number of hip fractures is expected to increase progressively over a calendar year with a greater than threefold increase by 2050 (Table 2).

**Table 2:** Estimated total number of hip and major osteoporotic fractures (MOF) in men and in women age 50 years or older in 2020 projected up to 2050 in Botswana

	2020	2030	2040	2050
*Hip fracture*				
Men	49	76	124	182
Women	54	78	119	190
Men and women	103	154	243	372
*MOF*				
Men	178	285	364	450
Women	217	314	471	655
Men and women	395	599	835	1105

Lifetime probabilities for hip fracture are shown in Table 3 for Botswana and selected countries. Lifetime risk was very low, substantially lower than Blacks from South Africa or the United States, and comparable to those reported for Tunisia.

**Table 3:** Lifetime probability of hip fracture in the Botswanan population from the age of 50 years compared with selected countries

Country	Lifetime risk at 50 years (%)	
	Women	Men
Sweden	25.6	11.0
South Africa (White)	23.4	7.7
Denmark	23.0	11.3
France	19.3	5.9
China (Hong Kong)	17.7	7.6
USA (Caucasian)	16.1	7.5
Turkey	15.9	3.6
Canada	15.5	5.8
Greece	15.4	6.8
Uzbekistan	14.7	8.7
UK	14.4	5.0
Germany	14.2	5.3
Portugal	13.7	4.8
Finland	12.9	6.0
Kazakhstan	12.6	6.0
Spain	12.6	4.2
Netherlands	12.5	5.4
Singapore (Indian)	12.5	5.2
South Africa (Indian)	12.1	4.6
Bulgaria	11.2	4.4
Hungary	10.8	4.2
Poland	10.1	4.2
Moldova	9.3	5.7
Kuwait	9.2	7.6
Abu Dhabi	8.9	8.1
Iran	8.3	5.5
Russia	7.7	3.8
Romania	7.0	3.8
South Africa (Coloured)	7.0	2.7
USA (Black)	5.9	2.7
Ukraine	5.6	2.9
South Africa (African)	4.5	1.9
Morocco	4.1	3.1
Botswana	1.1	1.4
Tunisia	0.7	0.7

## Discussion

This study documented the incidence of hip fracture in the Republic of Botswana. In both sexes, the incidence increased with age. Overall, Botswana belongs to the low-risk countries for hip fracture for men and women [20]. It is of interest that hip fractures were nearly as frequent in men as in women, a feature that has been seen in other low-risk countries [20, 22]. The incidence of hip fracture in Botswana was very similar to that for Tunisia and sub-Saharan Africa. In contrast, the rates for Morocco, Blacks from South Africa, and Blacks from the US was higher than that for Botswana—an effect also reflected in lifetime hip fracture probabilities.

Given that South Africa is a neighbouring country of Botswana, there is an interesting disparity between fracture incidence and fracture probability amongst Blacks from Southern Africa. Hip fracture rates in Botswana were approximately twofold lower than in Blacks from South Africa. Variations of this magnitude are seen within counties with much greater heterogeneity between countries [20]. In contrast, the difference in lifetime fracture probability in women was much larger (greater than fourfold different). The larger difference is due to the much lower mortality in Blacks from South Africa [23]. These observations emphasise the importance of the death hazard in the determination of fracture probability.

The number of hip fractures nationwide was estimated at 103 in 2020. Although fracture probabilities were low, demographic projections indicate that fracture burden is set to increase markedly in the future given the increase in the aged population. It is estimated that the annual number of hip and other major osteoporotic fractures will increase more than threefold over 30 years. The prediction is relatively robust in that all individuals who will be aged 50 years or more in 2050 are currently adults. However, these estimates may be conservative since they assume that the age- and sex-specific risk of hip fracture remains unchanged over this period. If the age- and sex-specific incidence of hip fracture increases, as has been registered in several countries [24], then the number of fractures may be more than doubled [25]. Such projections are important for healthcare planning.

Despite the training of dedicated research assistants and that fractures were treated only at specified hospitals, it is possible that cases were missed or did not come to hospital attention. The nature of the study did not permit an assessment of accuracy. Some patients may have been treated outside the country without the use of the public health system or medical insurance, while some may have been treated solely by traditional healers and thus not accounted for in the present study. Nonetheless, we think that such numbers are minimal, and we have thus captured the vast majority of hip fractures. In some countries, particularly in Eastern Europe, patients are not hospitalised because facilities for surgical management are limited so that hospital admission is not feasible [26–29]. There are additional limitations to this study. These include its retrospective nature and the relatively small sample size and, therefore, wide confidence intervals from which to compute fracture incidence. Although we avoided double counting, we included hip fractures irrespective of the level of trauma and those that may have been attributed to neoplastic disease. We were unable to stratify by HIV status because of too much missing data and could not determine the impact of HIV on these rates.

In summary, the incidence of hip fracture has been determined for Botswana from a retrospective study covering 3 years. These data will be used to create a country-specific FRAX model for the Republic of Botswana.
